# Global health in the face of ‘permacrises’: why primary health care matters

**DOI:** 10.1017/S1463423625100819

**Published:** 2026-01-22

**Authors:** Lundi-Anne Omam, Almighty Nchafack Nkengateh, Camille M. Mba

**Affiliations:** 1 Reach Out Cameroon, Buea, Cameroon; 2 Cambridge Public Health Interdisciplinary Research Centre, Department of Psychiatry, University of Cambridge, Cambridge, UK; 3 Clinton Health Access Initiative, Yaounde, Cameroon; 4 Institute of Health and Allied Professions, School of Social of Sciences, Nottingham Trent University, UK; 5 Department of Public Health, Faculty of Medicine and Biomedical Sciences, University of Yaoundé 1, Yaoundé, Cameroon

**Keywords:** climate change, conflict, outbreaks, permacrisis, primary health care

## Abstract

The world faces an era of ‘permacrisis’, marked by overlapping challenges such as climate change, conflicts, economic instability, and recurrent disease outbreaks, which disrupt health systems and deepen inequalities. Primary Health Care (PHC) is vital for addressing immediate health needs and social determinants, fostering resilience, and promoting equity during such crises. This opinion piece highlights PHC’s unique role in ensuring essential services, reducing barriers to care, and integrating health with broader social and environmental policies. In conflict-affected and climate-impacted regions, PHC supports community resilience, promotes health equity, and adapts to systemic shocks. Investing in PHC infrastructure, empowering community health workers, early disease detection, promoting climate-adaptive health practices and delivering integrated care can advance health for all. PHC offers a sustainable pathway to resilient health systems capable of navigating the complexities of a rapidly changing world.

## Introduction

On the heels of recent successive and at times, concurrent health, economic, environmental, and political crises; such as conflicts, natural disasters, the energy crisis, climate crises, unplanned urbanization, inflation, the ongoing challenges posed by infectious diseases such as mpox, Marburg virus, and other emerging threats, as well as the rising burden of non-communicable diseases (NCDs) (Junior *et al*., 2020; Ngo Bibaa, 2020; Chiolero, 2023), the World Health Organization for Europe has acknowledged that the region is facing permacrisis (World Health Organization, 2022). Permacrisis could be defined as ‘an extended period of instability and insecurity’, which encapsulates the protracted crises that have left lasting scars on global health systems (Chiolero, 2023). These overlapping crises pose an unprecedented challenge to the resilience of societies, economies, and health systems worldwide.

Permacrises do not merely represent temporary upheavals but rather protracted and concurrent crises that stretch health systems to their limits. The global health landscape faces an uncertain future and is evolving in response to these overlapping crises, creating new patterns of health challenges and exacerbating existing vulnerabilities (Sharma *et al*., 2025).

Despite significant progress in areas such as maternal and infant mortality, and immunization rates, the achievement of health for all remains a distant goal. For instance, global maternal mortality in 2020 was estimated at 223 deaths per 100,000 live births, with some countries seeing reductions, while others face stagnation or increasing rates (Moyer *et al*., 2023). Similarly, deaths from NCDs, which account for more than 70% of global deaths, are predicted to continue rising, particularly in low and middle-income countries (LMICs), where two-thirds of the deaths occur (Allen *et al*., 2021). Moreover, the emergence of infectious diseases like COVID-19, mpox and Marburg virus, alongside the continued risk of pandemics, has placed additional strain on already vulnerable health systems, highlighting the need for urgent investments in global health security (Hamdana *et al*., 2023; Han *et al*., 2023). For instance, global life expectancy dropped by 1.8 years in 2020 due to the pandemic, reversing gains made over the past decades (Chiolero, 2023).

## Why primary health care?

Primary Health Care (PHC) is often the first point of contact an individual has with the healthcare setting and can be delivered in different settings by a wide range of providers (World Health Organization, 2018a). Behera *et al*. (2022) describe PHC as the practice of diagnosing and managing health conditions, while also providing ongoing care and support for long-term and chronic illnesses. PHC offers an integrated, people-centred approach to health that addresses the root causes of poor health, including social determinants like poverty, inequality, and inadequate access to resources (World Health Organization, 2020). Unlike secondary and tertiary care, which tend to focus on specialized, acute care, PHC emphasizes prevention, early detection, and ongoing care at the community level (World Health Organization, 2019). PHC is well-suited to address the challenges posed by permacrises because it integrates health services with social and environmental policies, enabling a holistic approach to health that emphasizes resilience and equity (World Health Organization, 2018b).

Its primary aim is not only to treat illness but to prevent it and promote overall well-being (World Health Organization, 2020). In an era where permacrisis is a defining feature of global health, PHC is more important than ever. It provides a solid foundation for health systems to respond to multiple emergency situations such as the ongoing outbreaks of emerging infectious diseases, the long-term challenges posed by climate change, the negative health outcomes resulting from armed conflicts, and NCDs (World Health Organization, 2018b, World Health Organization & United Nations Children’s Fund, 2020).

PHC’s value lies in its ability to address both immediate health needs and long-term resilience. It ensures that essential health services remain accessible during times of crisis, whether caused by climate-related disasters, emerging infectious diseases, or political instability (World Health Organization, 2018b, Ngo Bibaa, 2020). For example, in conflict-affected settings of Ekondo-titi in Cameroon where communities were cut off from immunization services for over 5 years, a measles and Mpox outbreak were successfully contained with PHC interventions (Metuge *et al*., 2021, Jarman *et al*., 2022). Similarly, during the COVID-19 pandemic in Nigeria, adequate PHC staffing, flexible financing, and strong local government leadership were pivotal in restoring health service delivery volumes (Neill *et al*., 2023) The adaptability of PHC is further demonstrated by Aljohani *et al*. (2024), who highlight the role of general and family physicians as frontline providers offering immediate care, psychosocial support, and community leadership across multiple crises. In Europe, Kumpunen *et al*. (2022) describe the re-orientation of PHC systems into three distinct models: the deployment of multidisciplinary primary care teams for emergency response, the prioritization of vulnerable patients, and the adoption of digital tools to enhance both COVID-19 and non-COVID-19 care. These examples highlight PHC’s suitability for responding to public health emergencies, including the current global concern around mpox outbreaks (Zuluaga-Gómez *et al*., 2022). Moreover, the ongoing threat of future pandemics or other public health emergencies of international concern further emphasizes the need to invest in robust PHC systems capable of early detection, rapid response, and continuous care. While PHC is not a panacea for all crises, it offers a vital foundation for health systems, especially in low-resource and crisis-affected settings.

## Addressing systemic health challenges

A key contribution of PHC in an era of permacrisis is its capacity to address the underlying social determinants of health, including those exacerbated by climate change and conflict. Issues such as food insecurity, poor access to clean water, education, and housing are intricately linked to health outcomes (Marmot *et al*., 2008) and are increasingly threatened by environmental changes, such as droughts, floods, and extreme weather events, infectious disease outbreaks, as well as by the displacement caused by conflict. These challenges disproportionately affect vulnerable populations, heightening the burden of disease and creating barriers to accessing essential health services (Quinn and Kumar, 2014; Workman *et al*., 2022). By integrating health services with efforts to improve these social determinants, PHC can help mitigate the negative impacts of conflicts, climate change, disease outbreaks and food insecurity, promoting long-term resilience and better health outcomes in affected communities.

In conflict-affected settings, PHC offers a vital opportunity to bring services closer to populations, reducing the risks and challenges associated with long-distance travel to access stand-alone services. By delivering an integrated package of care, healthcare providers including Community Health Workers (CHWs) can ensure that communities receive comprehensive support while minimizing the risks of kidnapping or exposure to crossfire during travel (Omam and Metuge, 2023).

In the context of climate crises, PHC is essential in responding to the health impacts of extreme weather events, environmental pollution, and resource scarcity (Lokotola *et al*., 2023; Haines *et al*., 2024). The health effects of climate change, including the spread of vector-borne diseases, heat stress, and malnutrition, disproportionately affect vulnerable populations. PHC can play a critical role in building community resilience to these effects by providing health education, promoting climate-adaptive health practices, and ensuring access to healthcare in areas impacted by climate-related disasters.

During periods of disease outbreaks like the COVID-19 pandemic, health systems globally were stretched beyond their limits. The pandemic highlighted the critical need for enhanced primary health systems capable of managing routine care while responding to emergencies. PHC serves as the first line of defence, offering continuity of care for chronic conditions, mental health support, maternal and child health services, and vaccines, all essential elements in times of crisis (World Health Organization, 2020). These lessons are particularly pertinent as the world shifts its focus from COVID-19 to other emerging health threats, such as the resurgence of mpox, Marburg and the rising impact of new infectious outbreaks.

In the context of food insecurity, PHC can directly address malnutrition and its associated health impacts through integrated services such as community-based nutrition programmes, supplementary feeding initiatives, and micronutrient supplementation (Kraef *et al*., 2020; Omam and Metuge, 2023). For example, PHC facilities can screen children for malnutrition, provide therapeutic foods, and monitor their recovery, as seen in emergency nutrition interventions in drought-affected regions. Additionally, PHC can promote household food security through health education programmes on climate-resilient agriculture, breastfeeding practices, and diversified diets. Collaboration with local and international partners can further enhance these efforts by integrating food distribution and healthcare services, ensuring that populations receive both immediate nutritional support and long-term health benefits.

## A people-centred approach to permacrises

In the face of permacrises, a people-centred approach to health becomes indispensable. PHC stands out for its emphasis on community involvement and tailored care that addresses the multifaceted challenges these crises present. By engaging communities in decision-making and designing interventions that respond directly to their needs and specificities in the face of compounding challenges. For example, in conflict zones, PHC can deploy mobile clinics to reach displaced populations, while in areas affected by climate change-induced food insecurity, it can integrate nutritional screening and therapeutic feeding into routine services (Omam *et al*., 2021; Omam and Metuge, 2023). Such adaptability is critical in addressing the compounded barriers people face in permacrises, including long distances to care, financial constraints, and poorly equipped health facilities.

Moreover, by incorporating local knowledge and practices into health systems, PHC not only improves service delivery but also fosters trust and resilience within communities. Its flexibility facilitates effective operation in diverse settings, whether remote villages devastated by floods, overcrowded refugee camps, or urban slums grappling with disease outbreaks (Gizaw *et al*., 2022). A people-centred approach ensures that health interventions are not only immediate and responsive but also capable of strengthening global health security by enhancing community resilience, facilitating early detection of emerging diseases, and maintaining routine care during emergencies.

## Looking ahead: the role of PHC in shaping the future of global health

As the world faces an uncertain future marked by ongoing permacrisis, PHC remains a crucial pillar for advancing global health. The path to achieving universal health coverage and improving health outcomes in the face of global crises lies in strengthening primary health systems. PHC offers a holistic, inclusive, and sustainable approach to health that can mitigate the adverse effects of permacrisis while promoting long-term resilience (Chotchoungchatchai *et al*., 2020; World Health Organization & United Nations Children’s Fund, 2020).

To ensure that PHC can respond effectively to the challenges posed by permacrisis, governments, international organizations, and other stakeholders must invest in strengthening PHC systems, particularly in vulnerable settings. This includes increasing funding for health infrastructure, supporting the integration of CHWs in formal health systems, and ensuring that PHC services are integrated with broader social policies. The future of global health depends on a commitment to PHC as a cornerstone of sustainable health systems capable of withstanding the shocks of an increasingly uncertain world.

In conclusion, PHC is not merely a response to individual health crises; it is the cornerstone of global health security and resilience in an era defined by permacrisis. By investing in PHC, we can address the immediate health needs of populations, build long-term resilience to systemic and natural shocks, and tackle the root causes of health inequalities. Strengthening PHC is essential to navigating the complexities of permacrises and advancing toward the goal of achieving health for all, even in the face of persistent and interconnected global challenges.

## Data Availability

Not applicable.
